# Alcohol-Based Hand Sanitizers in COVID-19 Prevention: A Multidimensional Perspective

**DOI:** 10.3390/pharmacy9010064

**Published:** 2021-03-19

**Authors:** Kennedy Abuga, Nasser Nyamweya

**Affiliations:** 1 Department of Pharmaceutical Chemistry, School of Pharmacy, University of Nairobi, Nairobi P.O. Box 19676-00202, Kenya; 2 Pharma Manufacturing Solutions, Nairobi P.O. Box 21297-00505, Kenya; nasser04@yahoo.com

**Keywords:** hand sanitizers, alcohol, hand hygiene, tetrahedron, antiseptic, COVID-19

## Abstract

The global use of alcohol-based hand sanitizers (ABHS) as an important means of controlling the transmission of infectious disease has increased significantly as governments and public health agencies across the world advocated hand hygiene as a preventative measure during the COVID-19 pandemic. Although the performance of these products is most commonly defined as a function of their alcohol concentration, they are multifaceted products in which an interplay of several factors is important in determining efficacy. This paper discusses the interplay between ABHS input (formulation) factors and output (product performance) factors in the context of a multidimensional perspective using a novel representative paradigm. In the model, represented in the form of a three-dimensional tetrahedron, each of the faces represents inputs in the manufacturing of the ABHS product, which are the type and amount of alcohol, the inactive ingredients, the formulation and the manufacturing practices. The four corners of the tetrahedron represent the product performance factors which include product efficacy, sensory characteristics, usage and compliance and product safety. The multidimensional approach to the formulation and evaluation of ABHS shows that several factors contribute to the effectiveness and utility of these products. The paradigm provides a useful framework for manufacturers of ABHS and related healthcare products.

## 1. Introduction

Alcohol-based hand sanitizers (ABHS) have emerged as an important tool in the fight against SARS-CoV-2, the virus that causes coronavirus disease 2019 (COVID-19) [1]. The disease has spread rapidly throughout the world thereby necessitating stringent measures and controls to minimize its transmission. One of the key measures that has been advocated is the need to ensure hand hygiene [2].

Hand sanitizers are products that are applied and rubbed on hands to inactivate pathogenic microorganisms. These products are designed to dry rapidly after application, thereby eliminating the need for soap, water and drying aids such as towels. The convenience and portability of hand sanitizers has led to their widespread usage in 2020 as ABHS were recommended by the World Health Organization (WHO) as an alternative hand hygiene measure [3].

Ethanol and isopropanol (2-propanol) are the commonly used alcohols in ABHS. They are typically formulated as aqueous mixtures with several other ingredients such as emollients, moisturizers and fragrances. Although the major focus of ABHS performance has been the alcohol concentration, added ingredients and auxiliary factors play a critical role in their efficacy, safety and long-term utility.

**Figure 1:**
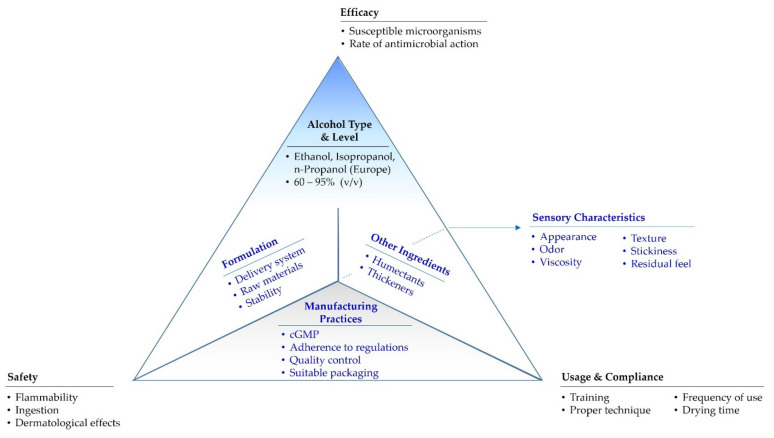
The alcohol-based hand sanitizers (ABHS) tetrahedron of manufacturing inputs and performance factors. Factors represented by the hidden faces and vertex are shown in blue font. Abbreviation: cGMP—current good manufacturing practices.

In this paper, the factors influencing the attributes of ABHS form a new multidimensional paradigm which is conveniently illustrated in the form of a tetrahedron (Figure 1). In this model, each of the faces of the tetrahedron represents inputs in the manufacturing of the product, i.e., (1) the type and amount of alcohol, (2) inactive ingredients, (3) the formulation, and (4) manufacturing practices. The four corners of the tetrahedron represent the product performance factors: (1) efficacy, (2) sensory characteristics, (3) usage and compliance and (4) product safety. While these factors are often considered individually, the long-term use of ABHS requires that manufacturers of these products address the manner in which the factors relate to each other and are integrated to provide a quality product.

## 2. Faces of the Tetrahedron: Inputs

The principal ingredients in ABHS are an alcohol (or mixture of alcohols) and water. Additionally, ABHS may have other ingredients which perform a variety of functions (Table 1). The influence of these ingredients on product efficacy, safety and usage must be considered. The WHO has developed two formulations based on either ethanol (80% *v*/*v*) or isopropanol (75% *v*/*v*) with glycerol (1.45% *v*/*v*) and hydrogen peroxide (0.125% *v*/*v*) [4]. Kratzel et al. demonstrated that WHO based formulations had broad spectrum antimicrobial activity including efficacy against SARS-CoV-2 [5]. Firms which manufacture commercial ABHS on a regular basis typically use proprietary formulations.

### 2.1. Alcohol Type and Level

The active ingredient in ABHS is an aqueous alcohol solution in optimized concentrations. Alcohols are known to possess broad-spectrum antimicrobial activity against bacteria, fungi and viruses. While the specific mechanism of action of alcohols is not clear, it is thought to be related to denaturation of membrane and enzymatic proteins [6,7,8].

Three alcohol homologs are utilized in ABHS namely, ethanol, isopropanol and n-propanol. In the United States, however, the Food and Drug Administration (FDA) does not permit the use of n-propanol in ABHS [9,10].

From the foregoing, the two most widely used alcohols in ABHS formulations are ethanol and isopropanol. Isopropanol is more effective against bacteria while ethanol is favorably virucidal, which may be due to differences in polarity [6]. Amongst the usable alcohols, ethanol emerges as the most common choice since it is easily produced through fermentation and distillation. For the formulation of ABHS, pharmaceutical or food grade ethanol is recommended. The use of technical grade alcohol is typically not permitted due the high level of impurities. Nevertheless, due to the high demand for ABHS during the COVID-19 pandemic, special authorization to use these products may be granted provided the raw materials meet the acceptance criteria that would justify application. The FDA guidance specifies control of alcohol impurities such as benzene, methanol, acetaldehyde and acetal to levels below specified limits. Non-compliance with these limits necessitates further testing for specific residual solvents [9].

The efficacy of alcohol as an antiseptic is dependent on its concentration. The recommended alcohol concentration in ABHS is generally cited as 60–95% (*v*/*v*). However, the optimum level is nuanced and is not necessarily the same for ethanol and isopropanol. This recommended alcohol ranges permit the alcohol to interact with functional proteins thus inactivating them. The lower range (60–75%) readily denatures proteins while higher levels (>95%) cause coagulation of membrane proteins hence preventing the alcohol from entering the cell. Moreover, alcohol levels of above 80% may reduce the contact time due to volatility which undermines efficacy of the formulation as well as adverse skin tolerance [11].

It may be desirable to prefer a median alcohol concentration of 70–80% (*v*/*v*). Products formulated with alcohol levels close to the 60% lower limit risk the active ingredient content falling below the threshold during processing, transport, storage or use as constituent alcohols may evaporate. Post market surveillance reports by regulatory authorities in a number of countries have found some products with alcohol assays below threshold limits [12,13].

### 2.2. Inactive Ingredients

Key considerations with regards to the use of ingredients are their influence on ABHS efficacy, safety and usage. The most commonly added ingredients in commercial ABHS formulations are humectants, thickeners and fragrances. Humectants such as glycerol help counteract the drying effect of alcohols, which may otherwise adversely affect skin integrity, especially with frequent and long term ABHS use. Thickeners, are added to increase viscosity and facilitate application of ABHS by making them easier to handle and reducing spillage. They are most often polymers of acrylic acid and its derivatives such as carbomers [11].

The other commonly encountered ingredients in commercial ABHS products are aloe and tocopherols, which in addition to possible beneficial dermatological effects, may enhance marketing appeal. Denaturing agents, such as denatonium benzoate, sucrose octaacetate, isopropanol or triethyl citrate are added to deter ingestion of ABHS [9].

The effects of ingredients in ABHS formulations may be complex sometimes resulting in unintended outcomes. An interesting example is glycerol, the most frequently used humectant, intended to reduce dryness and irritation of the products. Although structurally a sugar alcohol with reported antimicrobial activity of its own at high concentrations [14], a number of studies have reported that glycerol can reduce the antimicrobial activity of ABHS [15,16,17,18]. In a study which used the ethanol-based WHO formulation, lowering the glycerol level to 0.5% (*v*/*v*) was recommended as an optimal balance between antimicrobial efficacy and dermal tolerability [18]. The reason for glycerol’s effects on antimicrobial efficacy has not been elucidated, but may be related to its viscosity (which decreases the diffusion of the germicidal alcohol) when used above optimal levels. Since drying of the hands can be detrimental to skin integrity, the most commonly suggested solution has been to use a lower level of glycerol (as opposed to eliminating it completely).

Certain ingredients can potentiate antimicrobial ABHS activity. Organic acids, such as citric acid and phosphoric acid have been reported to increase the activity of ABHS against non-enveloped viruses [19,20,21,22].

Non-alcohol based hand sanitizers contain other antimicrobial agents such as benzalkonium chloride (BKC) [23]. Unlike alcohols, these ingredients are not volatile and hence antimicrobial activity can persist for longer periods [24]. Chlorhexidine is an example of an antimicrobial agent which has been combined with alcohol in some ABHS products [25,26] although its additional benefit has been questioned [27,28]. The use of such combinations is uncommon and may be restricted to ABHS employed in health care settings in some countries. Nevertheless, the use of non-alcoholic sanitizers in healthcare facilities is not recommended by the WHO.

An area which has not been extensively studied with ABHS is the potential for interactions between the various formulation ingredients. One reported example is that of chlorhexidine, which is cationic, with carbomer (an anionic polymer). Inactivation of chlorhexidine activity was observed in the presence of a carbomer containing ABHS [29]. There is also a potential for chlorhexidine to interact with anionic emulsifiers [30].

Additional potential interactions may arise from the presence of hydrogen peroxide (H_2_O_2_), a strong oxidizing agent which is a component of the two WHO recommended formulations [4]. The function of H_2_O_2_ is to inactivate bacterial spores as alcohols lack sporicidal action.

In good manufacturing practice (GMP) manufacturing environments, with more stringent control of raw materials, facilities and equipment, the use of H_2_O_2_ in the formulation is atypical. In cases where H_2_O_2_ is used, any ingredients that are susceptible to oxidation should not be included. Interactions between H_2_O_2_ and gel-forming polyacrylic acid polymers leading to a reduction in liquid viscosity have been reported [31]. In the study, polymer cross-link density, peroxide levels and the source/grade of H_2_O_2_ were shown to influence changes in viscosity.

The potential for ingredient–ingredient interactions and ingredient–container interactions will increase with the number of raw materials used in the formulation. Therefore, manufacturers should carefully consider the necessity of each added ingredient to the formulation.

### 2.3. Formulation

A number of delivery systems can be used for ABHS including liquids, gels, sprays, foams and wipes (Table 2). In hospitals and other public facilities, hand sanitizers are commonly packaged in dispensers. Low viscosity liquids and gels are the most common delivery modes for ABHS sold to the general public. Gels are essentially liquids with a significantly higher viscosity which assists in their application, especially from smaller containers. Free flowing liquids are better suited to delivery systems which have a higher degree of metering capability (e.g., dispensers, sprays and containers equipped with pump fittings).

Gels, along with foams, are preferred by many users due to greater ease of handling compared to low viscosity liquids [34]. Some studies, however, have suggested that the viscosity of gels may lower antimicrobial activity, possibly due to reduced diffusivity of the alcohol [35,36]. However, it appears that formulation factors such as increasing the concentration of alcohol may in such cases be used to improve efficacy [35].

The very different nature of other delivery formats makes comparisons challenging. Wipes or towelettes are a format that may be preferred by some people as they avoid the possibility of spillage that is associated with liquids or gels. A number of studies have compared different delivery systems. In one study, gel, foam and wipe ABHS delivery systems were comparable (no significant differences) in the reduction of virus counts on the hands [37]. Another study comparing a single-use ABHS packet that released product when folded to a single-use wipe revealed that patients in hospital or long-term care facility preferred the single-use packet to the wipe because they found it easier to handle [38].

### 2.4. Manufacturing and Packaging

In order to ensure that ABHS of suitable quality are produced, GMPs should be followed in their production. This includes the use of suitable manufacturing facilities, equipment, raw materials, systems, procedures, quality control testing, storage and distribution along with the requisite documentation and records.

Many countries have specific regulations that govern the production of ABHS [39]. In Europe, ABHS are regulated under the Biocidal Products Regulation [11,40] while in the United States, they are regulated as drug products by the FDA. Furthermore, the FDA distinguishes between health care and consumer antiseptic products [41].

Raw materials should meet the established standards for the countries in which the ABHS are marketed. For example, in the US, ethanol, isopropanol and glycerol should meet United States Pharmacopoeia (USP) or Food Chemicals Codex (FCC) specifications [9].

The safety of personnel involved in manufacturing and packaging operations is critical, especially given the potential of organic solvents to be flammable under uncontrolled conditions. Therefore, proper storage of alcohols and the use of air-conditioned and well-ventilated manufacturing rooms is necessary. Equipment grounding, the use of pneumatic mixers to prevent static discharge, lowering oxygen levels in mixing vessels and keeping alcohol vapor levels in the manufacturing rooms below threshold limits are examples of production safety measures that can be implemented [42].

While packaging may be sometimes considered secondary to the formulation of the ABHS, it is a critical component in ensuring product integrity, stability and delivery. ABHS are most commonly packaged in plastic containers. Container corrosion has been observed for the ethanol-based WHO formulation when packaged in aluminum beer cans [43].

Dispensers are commonly used for ABHS delivery in hospitals and other settings with high numbers of people. The design and function of dispensers are important in ensuring their effectiveness. In a study at a hospital which had recently installed dispensers, malfunctioning was found to be a common occurrence [44]. The need for regular auditing and maintenance of dispenser units has thus been stressed [45].

## 3. Corners of the Tetrahedron: Outputs

### 3.1. ABHS Efficacy

The efficacy of an ABHS is its key performance attribute. Depending on the application, target microorganisms and delivery mode, there are diverse standard test methods used to determine product efficacy [46]. Efficacy testing is generally performed using tests that measure the number of microorganisms before and after treatment with ABHS. Test results are typically expressed in terms of log10 reduction factors. The key factors influencing ABHS efficacy are the level and the type of alcohol used. However, added ingredients can also have significant influence on efficacy. For example, recent studies have shown that while the current WHO formulations do not meet the requirements for EN 1500 (hygienic hand rub) or EN 12,791 (surgical hand preparation), using higher alcohol levels (i.e., 80% ethanol or 75% isopropanol on a weight instead of the current volume basis) and lowering the glycerol level to 0.5% (*v*/*v*) enables these modified formulations to meet both EN test criteria [47].

### 3.2. Sensory Characteristics

The sensory characteristics of ABHS include the appearance, odor, viscosity, texture, skin feel, stickiness and residual feel of the product. Although sensory properties may not directly influence the efficacy of the product, they do play an important role in determining aesthetic appeal, which in turn determines the likelihood of individuals using a given product. Therefore, improving compliance may help reduce the transmission of infectious disease. The use of fragrances mitigates the intensity of the alcohol odor and also serves to distinguish various products, therefore making it an important manufacturing consideration.

Viscosity is a key property of ABHS as it influences factors such as dispensing, pourability, spreadability onto the skin and stickiness. Viscosity also influences drying time, which is an important consideration in user compliance.

The importance of sensory factors on compliance of ABHS has been studied [34]. Key desirable properties that were identified in the study included rapid absorption, a moisturizing hand feel, the absence of stickiness, a clean feel and the absence of or minimal odor. Gels have been reported to have better organoleptic properties than liquid formulations [36].

### 3.3. Use and Compliance

Proper product use is a critical component of the ABHS tetrahedron because even the best product will not be efficacious if used improperly. Key criteria in proper use of ABHS are the amount of product applied, the application time, the use of the proper technique and the drying time. Furthermore, application and drying times contribute to user acceptability and compliance. Multiple studies recommend that the minimum time required for hand rubbing is 15 s [48,49,50,51]. The benefits of instruction on ABHS use have been demonstrated with one study showing that training of health care workers on the proper technique resulted in improvements in both compliance and the effectiveness of hand sanitization [52,53].

Inadequate directions for use have been observed in many consumer ABHS products [54,55]. It is somewhat puzzling that manufacturers would invest time and resources into designing an efficacious product, yet to pay little attention to the importance of providing adequate directions to how users will apply the product. The need to view the product holistically is emphasized in the perspective of the tetrahedron. Improper use of ABHS will negate the ability of the product to properly disinfect hands. Additionally, the importance of readability and font size should be considered. This is especially a concern for small, portable ABHS bottle sizes.

ABHS users should be directed to apply amounts that are sufficient to wet the entire surface of the hands. Ideally, if the appropriate amounts of an efficacious ABHS are used, the applied product should dry rapidly so that the user can resume other activities. Long drying times, stickiness and residual effects are undesirable and may adversely influence compliance.

A study conducted among medical students demonstrated that reducing the six-step WHO hand hygiene technique to three steps yielded superior bacterial inactivation [52]. This proves that simplifying the hand hygiene procedure can achieve the desired results with improved adherence.

### 3.4. Safety

The main safety concerns with finished ABHS products at the consumer level are their flammability, ingestion (accidental or intentional) and potential adverse topical effects [56].

#### 3.4.1. Flammability

Alcohols are volatile and flammable organic solvents with flashpoints (lowest temperature at which emitted vapors can ignite) below room temperature. Ethanol-water mixtures at the operational concentrations in ABHS have flashpoints in the range of 17.5–22 °C [57,58].

The flammability risk poses fire hazards to ABHS users if these products are incorrectly used or stored. The ABHS may catch fire in the containers, during application or once applied onto users’ hands thus causing thermal injuries. A case of an individual who suffered burns from exposing hands wet with sanitizer to a flame illustrates this potential risk [59]. It should however, be noted that hazardous fires attributable to ABHS are uncommon. Nonetheless, it is essential to provide appropriate warnings concerning flammability on product labels, dispensers and storage areas.

#### 3.4.2. ABHS Ingestion (Accidental and Intentional)

ABHS, especially in liquid or gel packaged containers present ingestion risks [60]. ABHS products adulterated with methanol are especially concerning due to its higher toxicity. The intentional misuse of ABHS as a substitute for ethanolic beverages has led to serious adverse health consequences and deaths [61]. ABHS products may also be accidentally ingested by children [62]. In the US between 2011 and 2014 more than sixty-five thousand unintentional ABHS exposures in children aged ≤12 years were reported, the majority of which were by ingestion [63]. The FDA has also expressed concern about ABHS products with formulation (e.g., adding food flavors) or packaging (appealing coloring or markings) elements that may make them attractive to children [64].

Recently, the FDA has found methanol in several imported ABHS products in the US market [10]. The spike in demand for ABHS products occasioned by the COVID-19 pandemic, created an opportunity for substandard products to enter the market [65]. One of these products had a methanol concentration of 81% [66]. This highlights the importance of manufacturers ensuring that they have adequate quality controls to prevent adulterated products from reaching the market. In some cases, methanol is used as an ethanol substitute by ABHS manufacturers if it is more readily available or less costly than the permitted alcohols. Tragically, such actions resulted in hundreds of deaths and numerous cases of loss of sight in Iran in 2020 when individuals unknowingly consumed ABHS tainted with methanol [67].

The need for ABHS manufacturers to avoid methanol and methylated spirits containing methanol is critical [68]. Notably, the addition of denaturing agents to ABHS is a critical aspect of reducing the risk of their oral ingestion. Furthermore, stringent regulatory oversight is required to monitor ABHS manufacturers and supply chains [12].

#### 3.4.3. Topical Effects

The tolerability of ABHS is listed as one of the six golden rules of hand hygiene [69]. Even though, the dermal tolerability of an ABHS does not affect efficacy, adverse skin effects will most likely have a negative effect on usage and compliance [70]. Frequent application of skin irritants and allergens can result in contact dermatitis [71]. Generally, ABHS have been considered to have a low incidence of adverse dermal effects except for, in some cases, drying of the skin [72,73,74]. Application of moisturizing creams if ABHS are frequently used has been suggested [75]. Alcohols show a low potential for contact dermatitis although they may cause a burning sensation if they are applied to irritated or damaged skin [76,77]. The incorporation of emollients in an ABHS was reported to reduce contact dermatitis in a controlled, randomized, double-blind trial [78].

Recently, a specific case was reported in which a 12 year-old child developed contact irritant dermatitis following the over-application of a 70% isopropanol-based hand sanitizer [79]. Ethanol has been reported to be capable of causing contact dermatitis, although the causative chemical may be not necessarily be the alcohol but associated impurities or the aldehyde metabolite [80]. The potential of isopropanol to cause contact dermatitis has also been reported [81].

Tocopherol, fragrances, propylene glycol, benzoate, and cetyl stearyl alcohol have been reported as common potential allergens in ABHS used in the United States [82]. Fragrances in particular are a frequent cause of contact allergies in personal care products [83]. While some fragrance compounds may themselves be weak sensitizers, the oxidation of parent compounds may result in the generation of potent allergens [84]. ABHS users with specific allergen sensitivities are advised to review ABHS product ingredient lists and use low-allergen sanitizers [82].

A rise in frequency of hand eczema resulting from increased hand hygiene measures has been reported [85,86,87] although these reports did not indicate whether this was due to increased hand washing or ABHS use. In one reported case, a patient developed palmar erythema due to excessive use of ABHS. The condition resolved soon after the use of the ABHS was reduced [88].

Another related potentially adverse topical effect that has been reported in the literature recently is accidental ocular exposure to ABHS [89,90,91]. It is therefore important that ABHS users be made aware of this risk and use by children be carefully monitored by an adult. Preventative measures for minimizing ocular exposure while using ABHS have been proposed [92].

The high use of ABHS during the COVID-19 pandemic has also been reported to be a possible cause of disulfiram alcohol reactions [93,94]. However, it has been proposed that inadvertent inhalation (especially if ABHS are used in poorly ventilated areas) rather than topical absorption is more likely to result in high enough amounts of alcohol exposure to elicit a disulfiram reaction [95].

## 4. Model Paradigm

The proposed hand sanitizer model paradigm captures the relationships between ABHS product inputs and outputs. For example, the previously mentioned approaches that have been used by a number of researchers to improve the efficacy of the WHO formulations reveal an interplay between alcohol level and added ingredients (e.g., glycerol) that influences product efficacy. The counteracting influence of humectants on the drying effects of alcohols show how potential adverse effects on the integrity of the skin by ABHS can be mitigated. ABHS delivery formats are a contributing factor to the use of ABHS by individuals who may exhibit preferences for a specific type of product. Delivery systems and sensory characteristics of ABHS play an important role in making these products more attractive to users which is a critical component of compliance. While it may be argued that sensory characteristics do not play a role in efficacy, it is quite clear from the widespread use of fragrances and coloring agents in commercially marketed ABHS products that they do influence customer appeal which indirectly relate to usage and compliance.

The majority of ABHS studies have been performed in hospital environments or controlled settings. The challenge though in the current COVID-19 environment is that ABHS are being widely used by the general public in a multitude of products and delivery formats. With the tremendous increase in ABHS use globally product factors such as ease of use, convenience, portability, user preferences, how well users follow directions for use and safety will become increasingly important. The evaluation of the quality attributes of ABHS products will therefore need to encompass a range of factors that are illustrated in the ABHS tetrahedron paradigm.

## 5. Conclusions

This review highlights the need for ABHS to be viewed from a multidimensional perspective from the design level as the factors that go into their formulation impact product attributes and performance. It is important to recognize the interplay of these factors in order to ensure ABHS product efficacy and safety. The review also emphasizes the following:

- ABHS are the method recommended by the WHO for ensuring hand hygiene to curb the spread of the COVID-19 pandemic due to superior efficacy and convenience.
- ABHS need to be carefully designed and formulated for the desired quality, efficacy and safety.
- Inactive ingredients used in ABHS may have unexpected effects on product performance.
- Safety features should be designed into ABHS products to minimize risks such as flammability and exposure by ingestion.

## Figures and Tables

**Table 1:** Ingredients commonly used in commercial ABHS products.

Ingredient	Functions	Examples ^†^
Active (alcohol)	Inactivate susceptible microorganisms	Ethanol, Isopropanol, *n*-propanol ^‡^
Solvent/Cosolvent	Facilitate alcohol protein denaturation Reduce product volatility	Water
Humectant	Facilitate skin hydration	Glycerol, Propylene glycol
Emollient	Maintain skin softness, smoothness, pliability	Caprylyl glycol, Isopropyl myristate
Thickener	Increase viscosity, facilitate handling; reduce spillage/runoff	Carbomer, Acrylates/C10-30 alkyl acrylate cross polymer
pH adjusting agent	Neutralization of acrylic acid-based polymers to enhance viscosity	Aminomethyl propanol, Triethanolamine
Fragrance	Enhance aesthetic appeal, ameliorate/mask alcohol odor	Linalool, Limonene
**Others**: Antioxidant Multifunctional	Ameliorate adverse effects of alcohols on the skin	Tocopheryl acetate Aloe vera

^†^ Some ingredients may have more than one or overlapping functions. ^‡^ *n*-propanol is not approved for use in the USA.

**Table 2:** ABHS Delivery Formats.

Formulation	Delivery System	Advantages	Disadvantages
Liquid (low viscosity solutions)	Pouring/squeezing from bottles Pumping from containers Dispensers	Portable Widely available	Spillage, dose metering Alcohol odor may be more pronounced
Gel	Pouring/squeezing from bottles Pumping from containers Dispensers	As for liquids, but with better dose metering, handling characteristics	Spillage, antimicrobial action may not be as rapid as that of liquids Longer drying time than liquids
Foams	Dispensers Special containers	As for gels, but with reduced spillage May be preferred by some consumers	More expensive than liquids or gels
Dispensers	Mechanical (lever) Touchless (sensor)	Controlled dose metering for liquids, gels and foams	Device malfunctioning will prevent dosing Potential fomite risks [32,33]
Sprays	Actuation of a valve	Controlled dose metering	Product losses to atmosphere Potential for inhalation Higher flammability risk if incorrectly used
Wipes	ABHS is transferred from wipe to surface of the skin	Dose metering Portable, convenient, no spillage	Needs to be designed to provide sufficient amount of ABHS in each wipe Non-biodegradable fabrics
